# *Rhizobium rhizogenes* infection in threatened Indian orchid *Dendrobium ovatum* mobilises ‘Moscatilin’ to enhance plant defensins

**DOI:** 10.1007/s13205-022-03180-9

**Published:** 2022-04-23

**Authors:** Ipsita Pujari, Vidhu Sankar Babu

**Affiliations:** Department of Plant Sciences, Manipal School of Life Sciences, Manipal Academy of Higher Education, Manipal, Udupi, Karnataka 576104 India

**Keywords:** Defence mechanism, *Dendrobium ovatum*, Moscatilin. Phenanthrene, *Rhizobium rhizogenes*

## Abstract

The present study illustrates the transformation ability of two wild-type bacterial strains of *Rhizobium rhizogene*s (MTCC 532 and MTCC 2364) on the embryogenic callus and callus-derived plantlets of a threatened Indian orchid, *Dendrobium ovatum*. Co-culture of the bacterium with the explants gave marginal hairy root phenotype that failed to multiply in the culture medium. Some primary and secondary metabolites were subdued in infected explants. Moscatilin, the stilbenoid active principle in *D. ovatum,* was found below the detection limit. The presence of two metabolites viz., Laudanosine, a benzyltetrahydroisoquinoline alkaloid and Lyciumin B, a cyclic peptide, were detected exclusively in the infected explants. The subjugated amino acids and phenolics in the infected plantlets were routed to produce phytoanticipins, and phenanthrenes, strengthening the defence mechanism in infected tissues. This research implies that the plant's defence mechanism activation could have prevented the extensive hairy root formation in the explants, even though nodulations and phenotype transitions were witnessed. Moscatilin has a structural resemblance with Resveratrol, a phytoalexin that combats bacterial and fungal pathogens. The study favours the possibility of Moscatlin being a precursor for phenanthrene compounds, thereby serving as a ‘phytoanticipin’ during the infection phase.

**Supplementary Information:**

The online version contains supplementary material available at 10.1007/s13205-022-03180-9.

## Introduction

Micropropagation of ornamental plants has always been centered towards germplasm conservation through rapid mass production and development of new cultivars via recombinant DNA techniques (Teixeira da Silva et al. 2020). In ornamentals, the intense focus has always been on developing disease-free, viral-resistant cultivars with superior traits such as high-throughput production of phytochemicals. Several in vitro strategies have been adopted to enrich plant natural products, but ‘hairy root culture,’ i.e., ‘*Rhizobium rhizogenes*-mediated transformation’ in plants, holds much significance in its productivity competency and steadiness (Chandran et al. 2020).

The study species, *Dendrobium ovatum,* is a threatened tropical epiphytic orchid, which is endemic to the Western Ghats, India. It encompasses therapeutically significant compounds, including the stilbenoid ‘Moscatilin’, an anticancer therapeutic and one of the distinguished active compounds in *Dendrobiums* (Lai et al. 2020). Production of this phytochemical in the wild is affected by many confounding factors, including environmental disruptions. Chemical synthesis of Moscatilin and the closely related stilbenes are often complex due to the non-availability of suitable starting materials, and hence plants are utilised as natural producers. Considering the threatened or endangered nature of most *Dendrobiums*, avoiding the environmental cues is essential to stabilise the production of low-produced medicinally and commercially important compounds, which is why tissue culture approaches play a vital role in this context. In view of enhancing the essential phytocompounds, the axenic cultures have been tested immensely towards their genetic transformation ability using *Agrobacterium rhizogenes* (currently addressed as *Rhizobium rhizogenes*) (Boobalan et al. 2020). Hairy roots offer cellular differentiation, which is why it is more feasible to augment metabolites through the pathway regulations that prefer or require cellular compartmentalisation for their accumulation (Ge and Wu 2005). Hairy root culture is reliant primarily on the regeneration competence of the explants. In this study, a regeneration platform was established via the formation of protocorms and protocorm-like bodies (PLBs) that gave rise to embryogenic and organogenic calluses, which regenerated into plantlets. The ability of *D. ovatum* to produce hairy roots through *R. rhizogenes* transformation was monitored in the present study to harvest a high-value plant product, Moscatilin. Along with Moscatilin, other metabolites were also analysed during the infection phase.

*Rhizobiums* are identified as keystone taxa ubiquitously found even in extreme ecosystems such as Artic Antarctic locales, contaminated soils, and forests, closely associated with the plants (Altaf 2021; Díez-Méndez and Menéndez 2021; Moriya et al. 2021; Otten 2021; Padilla et al. 2021; Turgut and Basim 2021)*.* Hairy root cultures have been successful in upscaling natural products of therapeutic significance such as Camptothecin (Vincent 1981), Artemisinin (Putalun et al. 2007), Verbascoside (Dhakulkar et al. 2005) and Scopolamine (Jouhikainen et al. 1999). It is also beneficial in accumulating compounds that are usually below the detection limit in the stock plants (Bahmani et al. 2021).

The current study examined the transformation ability of two wild-type bacterial strains of *Rhizobium rhizogene*s (MTCC 532 and MTCC 2364) on the embryogenic callus and callus-derived plantlets of a threatened Indian orchid, *Dendrobium ovatum* and its ability to generate hairy roots. We checked the metabolite transitions during the infection phase, notably the active principle Moscatilin. Since, Moscatilin has a structural resemblance with Resveratrol, a phytoalexin that combats microbes; it would be interesting to find the role of Moscatilin during infection and transformation phases.

## Materials and methods

### Callus induction and regeneration

**Fig. 1:**
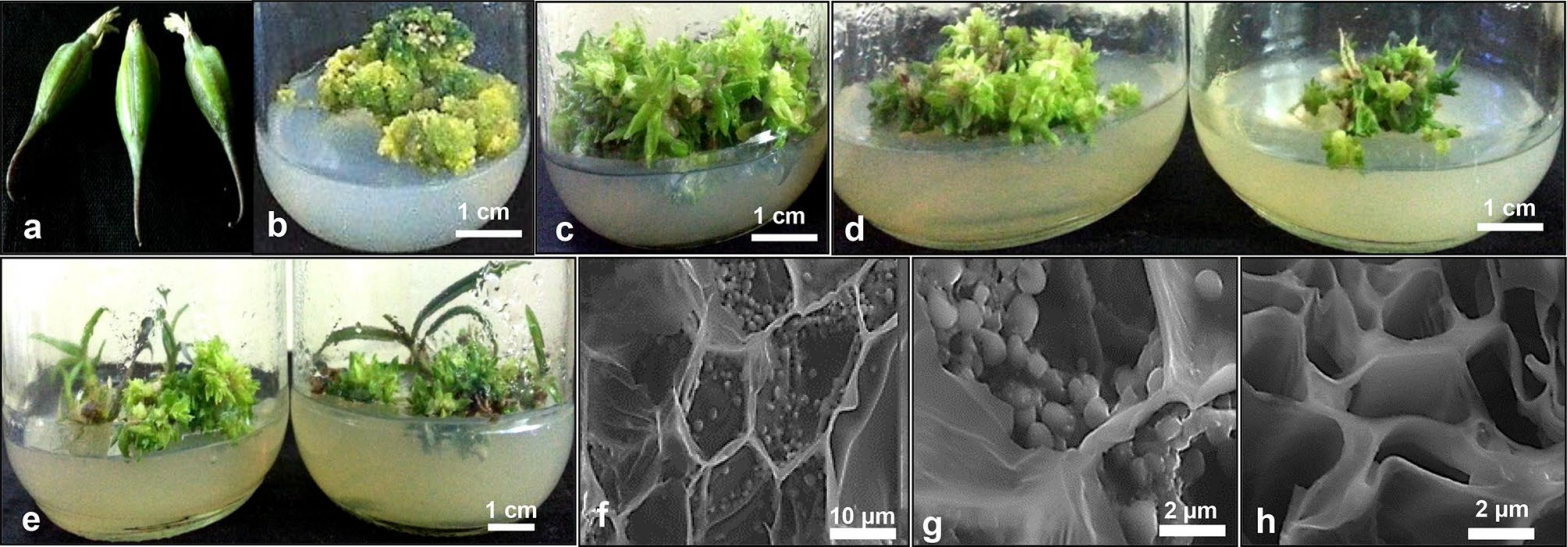
**a** Capsules of *Dendrobium ovatum*. **b** Induction of callus. **c** Organogenic callus transforming into plantlets on the 160th day of culture. **d** Callus-derived plantlets. **e** Callus-derived plantlets with well-developed shoots, shown at 200th day of culture. **f **, ** g** Infected tissues (callus-derived plantlets), showing nodulations in the cell walls. **h** Non-infected tissues (callus-derived plantlets), devoid of nodulations

Minute seeds of *D. ovatum* capsules were surface sterilised with 10% Tween 20 detergent followed by 0.1% mercuric chloride (Fig. 1a), and the seeds were scooped out of capsules and were dusted onto half-strength Murashige and Skoog (MS) basal medium (Murashige and Skoog 1962) that was supplemented with 1 mg L^−1^ Zeatin hormone together with 2% (*w/v*) of Sucrose, and the pH was fixed to 5.8. A 12-h photoperiod per day was maintained for the *D. ovatum* cultures. All the cultures were kept at the temperature of 25 ± 1 °C, under cool fluorescent light provided through white fluorescent lamps at 40 μmol m^−2^ s^−1^. The protocorms and PLBs developed from the seeds were shifted to half-strength MS medium, augmented with 5 mg L^−1^ 2,4-D in conjugation with 3% (*w/v*) of Sucrose, where the pH was fixed to 5.8 towards the induction of callus. The subculturing was carried out every 20 days to achieve proliferation in callus. The regeneration of the calli was assessed after shifting to the ‘protocorms and PLBs induction medium’, i.e., half-strength MS medium solidified with Agar and augmented with 1 mg L^−1^ Zeatin and 2% (*w/v*) of Sucrose at pH-5.8.

### Raising *Rhizobium rhizogenes* cultures

Two different wild-type strains of *R. rhizogenes* (MTCC 532 and MTCC 2364) were collected from the Microbial Type Culture Collection and Gene Bank (MTCC), housed at the Institute of Microbial Technology Chandigarh, India. Reconstitutions of strains were done individually by adding 500 μl of freshly prepared Yeast Extract Beef (YEB) medium. The bacterial powder was diluted further by adding 200 μl of YEB medium. Four glass Petri plates and their replicates were prepared for streaking viz., plates deprived of antibiotic and bacteria (control), plates with only Rifampicin, plates with only Kanamycin A and plates containing both Rifampicin and Kanamycin A. *R. rhizogenes* strains were then streaked over the relevant Petri plates. After streaking, all the Petri plates were incubated in the dark at room temperature. Post 48 h, single colonies of bacteria were observed in the Petri plates that contained Rifampicin. *R. rhizogenes* suspension cultures were generated. Single bacterial colonies from the Rifampicin selection plates were mixed with freshly prepared and autoclaved YEB medium in a glass flask. Control (YEB medium with only Rifampicin) and test (YEB medium with both Rifampicin and bacterial colonies) were maintained separately during the bacterial suspension cultures. Both control and test flasks were placed on a rotary shaker, at a speed of 180–200 rpm (agitation) in the dark and kept at room temperature for about 48 h. Every 6 h OD (absorbance) readings were taken through Shimadzu UV-1800 Spectrophotometer at 600 nm for 48 h to estimate various phases during bacterial growth. Bacterial suspension with OD range between 0.4 and 0.8 (log/exponential phase) was picked for the infection with two different explant types of *D. ovatum* viz., callus and callus-derived plantlets, towards hairy or, transformed root culture.

### Co-culture of *D. ovatum *in vitro tissues with *R. rhizogenes* for hairy root induction

The first step of the infection involved wounding the plant tissues. These wounded tissues were dipped into the bacterial suspension, and they were kept with gentle shaking at 100–120 rpm for 3–4 h at room temperature to facilitate the bacterial invasion. For transformation, 36–42 h old (peak of exponential phase) cultures of *R. rhizogenes* strains were considered. First, they were centrifuged at 3,000 rpm for 5 min and then the resultant cell suspension was used towards infection. After 3–4 h of incubation, the plant tissues were dried through sterile filter papers and transferred to the culture bottles containing fresh and autoclaved MS liquid medium. This MS co-cultivation medium comprised 1 mg L^−1^ Zeatin and 2% (*w/v*) of Sucrose. For each explant type, control (non-infected) and test (infected) were maintained separately in different culture bottles in the dark at 25 ± 1 °C. After 3 days of infection, the infected explant types were removed from the co-cultivation media and washed thoroughly with the antibiotic Cefotaxime (500 mg L^−1^) to hinder excessive bacterial growth. Post wash, the explants were again transferred to the culture bottles with freshly prepared MS medium with 1 mg L^−1^ Zeatin hormone and 2% (*w/v*) of Sucrose with pH-5.8. Cefotaxime wash was continued till the bacterial growth subsided. Explants were continuously subcultured every 20 days and were monitored for the appearance of hairy root phenotypes in them. Various analyses viz., Stereo zoom (Motic), bright-field (Olympus BX51), and scanning electron microscopy (Leica) were carried out for the phenotypic analysis of hairy roots when they were during the 8th–10th week after co-cultivation.

## Metabolite screening

### Preparation of the extract

The best explant (callus/callus-derived plantlet) that generated a visual hairy root phenotype was considered for the metabolite profiling. While preparation of the extract, both non-infected (‘control’) and infected explants with hairy roots (as ‘test’) [1 gm. dry weight of each] were powdered using liquid nitrogen, and the fine plant powder was dissolved in the solvent methanol for extraction. This was subjected to sonication for around 45 min with an intermittent pulse of 30 s. Sonication was followed by centrifugation at 5000 rpm for 15 min at room temperature, and the supernatant was syringe filtered. The extract solutions were lyophilised then, and they were stored at – 20 °C till use.

### Analysis of the extracts through reversed phase-high performance liquid chromatography (RP-HPLC) and mass spectrometry (targeted and non-targeted metabolite profiling)

The lyophilised extracts were analysed using RP-HPLC and mass spectrometry. RP-HPLC instrument (Waters^®^ Alliance e2695 Separations Module) was furnished with an autosampler, degasser and ultraviolet detector (Waters^®^ 2487 Dual Wavelength Absorbance Detector) that was set at 281 nm wavelength along with a dual pump. RP-HPLC in the form of targeted profiling was carried out in this research to identify and quantify ‘Moscatilin’ (active principle), found in most of the *Dendrobiums*. Standard of Moscatilin was purchased from Chengdu Biopurify Phytochemicals Ltd., Sichuan, China and its preparation was done in 99.8% pure HPLC grade methanol at a concentration of 5 mg/ml. A set of working standard solutions (0.5, 1.0, 1.5 and 2.0 mg/ml) was also prepared freshly from the stock solution of Moscatilin using the solvent Methanol. Through RP-HPLC, the optimal separation was attained through isocratic elution mode using a mobile phase with the solvents viz., Acetonitrile (ACN)—eluent ‘A’ and 0.001% Trifluoroacetic acid (TFA) in milli-Q water—eluent ‘B’ in 40:60 (*v/v*) ratio. The solvents were degassed by sonication for 15–20 min at room temperature before use. Chromatographic separation was achieved on a C18 column at a temperature of 30 °C. While analysis, the pressure was maintained at around 1200–1300 psi, and the flow rate was 1 ml/minute. 10 μl (injection volume) of Moscatilin standard, blank and test samples were injected into the RP-HPLC instrument, and the run time for each sample was fixed to 30 min. The compound Moscatilin was identified in all the test samples by comparing its retention time with that of the authentic standard. The quantitative estimations were done in replicates (*n* = 5), and Moscatilin yields were represented as µg/g equivalents of the dry weight of *D. ovatum* tissue extract.

The mass spectrometric analysis was carried out in non-targeted metabolite profiling mode for callus-derived plantlets and their hairy root counterparts. The mass spectrometer was equipped with an electrospray ionisation source using the C18 column. A positive ion mode was used along with the following conditions; capillary voltage 19 V, spray voltage 5.0 kV, capillary temperature 300 °C, sheath gas flow rate at 40 (arbitrary units), the auxiliary gas flow rate at 20 (arbitrary units) and tube lens offset 40 V. The full scan EI mass spectra were acquired in the m/z range of 50–2000. 10 μl of the clear extract solutions (control and test) were injected into the carrier gas stream of the electrospray source. Mass spectra were retrieved, and the identification of all the compounds in the control and test systems was made based upon the mass, m/z and chemical formula, through the matching of the same in the METLIN database (within 5–15 ppm range), KEGG Compound database and PlantCyc database. Differences in the metabolites were observed between non-infected and infected explants in all the studied replicates.

## Results and discussion

**Fig. 2:**
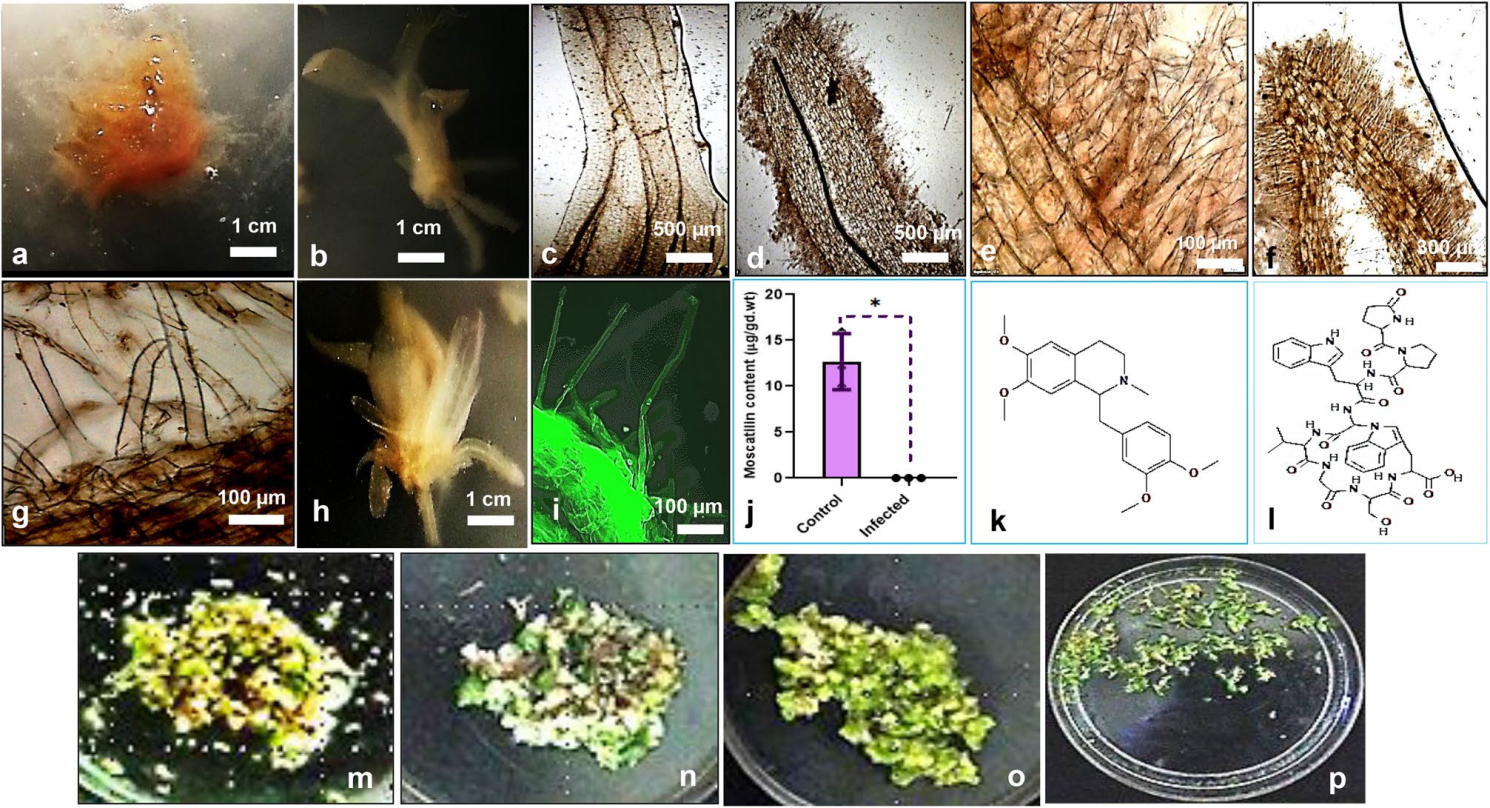
**a** Callus of *Dendrobium ovatum* co-cultured with *Rhizobium rhizogenes* wild-type strain MTCC 2364, shown after 8 weeks of co-cultivation. **b ** and ** h** Infected stems of callus-derived plantlets, showing adventitious (hairy) roots emerging from the base of the stem, shown after 8 weeks of co-cultivation. **c** Lateral sections of uninfected callus-derived plantlets of *D. ovatum* devoid of hairy roots. **d**–**g** Lateral sections of infected callus-derived plantlets of *D. ovatum,* displaying the emergence of hairy adventitious roots, shown after 4 weeks of co-cultivation. **i** Autofluorescence (under UV light) of transformed plant tissue i.e., callus-derived plantlet, showing the hairy roots formed. **j** Bar graph displaying the content of the active principle of *D. ovatum*, viz., ‘Moscatilin’ in control (non-infected) and infected samples, where the compound content was found below the limit of detection. Identification of two exclusive metabolites, viz., **k** Laudanosine, and **l** Lyciumin B in the infected callus-derived plantlets of *D. ovatum,* post-infection with *Rhizobium rhizogenes* wild-type strains. **m** coinfected callus. **n, o** denotes infected callus after washing with Cefotaxime on the 8th day and 12th day respectively. **p** denotes small plantlets emerging from the infected callus on the 28th day, proving that there was no bacterial regrowth in cultures after cefotaxime wash

Through *R. rhizogenes* transformation, callus and callus-derived plantlets underwent specific phenotypic transitions. Many research studies have indicated how the physiological status of explants plays a crucial role during the invasion of the bacterium (Mohammed et al. 2019). Callus and callus-derived plantlets have been chosen as explants towards hairy root culture in the present study, as many research reports have shown the transformation success during callus (embryogenic and organogenic) stages in many monocots (Mohammed et al. 2019; Koetle et al. 2015). Callus-derived plantlets have been considered because of their excellent regenerative capacity. The same has been considered the essential parameter in many studies involving monocot plants. These plantlets were found to be more accessible towards the process of transformation (Koetle et al. 2015). Tiny hairy roots started appearing on callus-derived plantlets after 4 weeks from the first day of co-cultivation (Fig. 2d–g, i). In non-infected explant types, no hairy root formation was observed (Fig. 2c). After around 8 weeks, in callus-derived plantlets, the hairy roots were more noticeable (Fig. 2b, h), whereas in calluses of *D. ovatum*, hairy roots formed were not much clear and observable (Fig. 2a). The calluses of *D. ovatum* obtained in the present study were rigid primarily. It was shown in rice plants earlier that the embryogenic calluses had reduced transformation abilities compared to the friable calluses, as they failed to treat plant growth regulator supplementations and media changes (Visarada et al. 2002). It was found that not all the infected explant types demonstrated the hairy root phenotypes, although there was a successful invasion of the bacterium into the plant cells as visualised through electron micrographs (Fig. 1i, j), unlike in non-infected explants (Fig. 1k). Molecular confirmation of ‘rol gene expression’ failed in the present study. After cefotaxime wash, the regrowth of the bacteria was not witnessed in cultures (Fig. 2m–p). Transformation frequencies exhibiting hairy root phenotypes in both the explant types were found to be ~ 40% through infection of the strain MTCC 2364, whereas it was limited to only ~ 20% through MTCC 532 infections.

Further growth of hairy roots with abundant multiplication was not witnessed in the MS co-cultivation medium. The observations from the transformation experimentation in *D. ovatum* indicated that, like many other monocot plants, this species of *Dendrobium* is also not a natural host of *R. rhizogenes* (Sood et al. 2011). Transformation (especially, *R. rhizogenes*-mediated) in monocots has not been much successful till date, and this is primarily due to the failure of the bacterium to reach competent cells and the inefficient regenerative capacity of the plants (Koetle et al. 2015). Also, it has been reported that the transformation via *Rhizobium* species is cumbersome in monocots because of its dependence on the plant genotype and cell wall chemistry. Unlike hydroxyproline-rich extensions in dicot plants, threonine-rich proteins have been witnessed in the cell walls during monocot cell differentiation (Sood et al. 2011). The meristematic cells of monocot plants generally lessen the *vir* gene functions, affecting the pathogenic strength of *R. rhizogenes*. And this happens because, at a very early developmental stage, cells lose their dedifferentiation capability and there occurs non-availability of certain metabolite exudates that function towards the induction of *vir* genes (Mohammed et al. 2019). During transformation, the first critical step is the attachment of *R. rhizogenes* to the plant's cell surface, which takes place through cell–cell recognition. The absence of appropriate receptors mostly leads to this attachment failure or low attachment due to improper binding. This causes the blockage of transfer (T)-DNA integration with root loci (*rol*) genes from the hairy root-inducing (Ri) plasmids of *R. rhizogenes* (Sood et al. 2011). Hairy root transformation studies in plants are usually centered on decoding the plant-pathogen interactions that appear in the form of either nodulation or mycorrhisation (Jain et al. 2020). If these are enabled in plants, it may reveal the underlying mechanism of hormone transport and rhizogenesis in monocot species. The results obtained through this research study demonstrated that an efficient *R. rhizogenes*-mediated transformation for threatened Indian orchid, *D. ovatum* is marginally attainable, and there are still a few impediments to cross. Further studies should be carried out to rightly understand the mechanism behind the bacterial invasion into the distinctive in vitro explant types of *D. ovatum* during transformation. This information can play a pivotal role in manifesting the completely successful transformation with T-DNA integration in recalcitrant monocots.

**Fig. 3:**
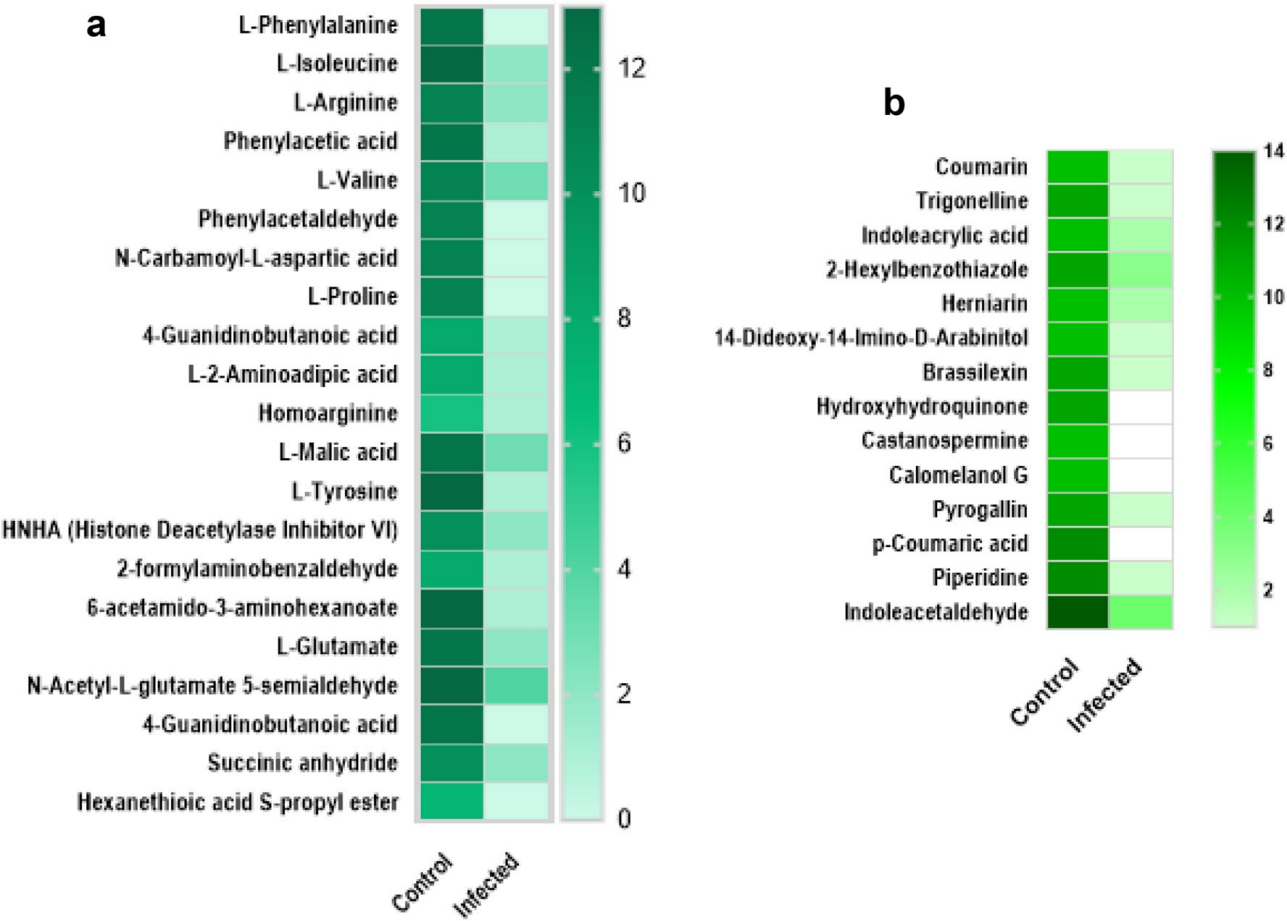
**a** Heat map showing the list of primary metabolites that were found subjugated in *Rhizobium rhizogenes* infected tissues of *D. ovatum.* **b** Heat map showing the list of secondary metabolites that were found subdued in the infected tissues of *D. ovatum*. The heat maps represent ∆ PPM values of metabolites in both infected and non-infected (control) samples

**Fig. 4:**
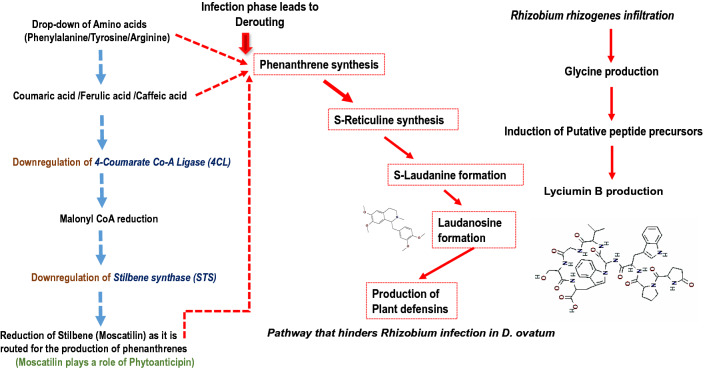
Plausible pathway (in red) that hinders *Rhizobium* infection in *D. ovatum*. Moscatilin is utilised for phenanthrene synthesis to produce plant defensins that resist infection, thus playing the role of a phytoanticipin. The usual pathway of Moscatilin (stilbene) synthesis (in blue) is downregulated. *Rhizobium* infiltration also enhances the production of cyclopeptide, notably Lyciumin B, which functions as an antimicrobial

Few research studies have reported that hairy root culture techniques with efficient biosynthetic potential are preferred over simple callus cultures to produce specialised phytochemicals with high biomass (Gutierrez-Valdes et al. 2020). This approach comprehensively serves as a perfect tool for studying the entire metabolism process in plant bodies by elucidating all the linked biochemical pathways. The untargeted metabolite analyses of cultures (both infected and non-infected) revealed that specific primary and secondary metabolites were subdued in *Rhizobium* infected samples (Fig. 3a, b). Tables S1, S2, S3, and S4 summarise the exclusive and common primary and secondary metabolites detected in infected and transformed callus-derived plantlets of *Dendrobium ovatum*. Primarily *R. rhizogenes* infection was found to interrupt the biosyntheses of essential amino acids viz., Tyrosine, Arginine and Phenylalanine and the intermediate compounds were found restrained in infected cultures. The active principle ‘Moscatilin’ was found below the limit of detection in infected sample tissues (Fig. 2j). The chromatogram of Moscatilin detected in RP-HPLC from the control sample (non-infected) is depicted in the supplementary information (Fig. S2). Many of the metabolites identified in infected regenerative plantlets were found crucial to evoke cell proliferation and regeneration (Fig. 3a, b). If channeled for playing the roles of plant defensins (metabolites that guard plants against pathogens), these metabolites may not be available for regeneration (Fig. 4). The reasoning above aligns with the present study as the *Rhizobium* infected samples decelerated regeneration capacity even though they exhibited marginal hairy root phenotype. The inability of *R. rhizogenes* to invade interior cells of the tissues could be due to the presence of specific metabolites, which may also cause cell-to-cell incompatibility impeding pathogen recognition by plant hosts (Walker et al. 2020). It is also possible that metabolites transform into different structures to guard the plant cells against pathogens.

Two exclusive metabolites were found in infected samples viz., Laudanosine, a benzyltetrahydroisoquinoline alkaloid, generally found in Poppy plants and Lyciumin B, a tryptophan-derived glycoside, commonly found in Solanaceae family. Laudanosine is involved in Papaverine biosynthesis (Pienkny et al. 2009). Papaverine functions as ‘phytoanticipins’ in plants activating defence mechanisms serving as defense alarms. The start point of Papaverine (a tropane alkaloid) synthesis is from L-tyrosine. The presence of Laudanosine indicated that the amino acids are often navigated towards the synthesis of phytoanticipins like Papaverine during the infection phase (Gutiérrez-Grijalva et al. 2020). Stilbenes and stilbenoids serve as precursors for the synthesis of phenanthrenes. In poppy plants, it is experimentally proven that aporphine types of alkaloids are generally synthesised from reticuline by phenol couplings (Morris and Facchini 2016). Radioactive norlaudanosoline^155^ revealed that the synthesis of certain phthalide isoquinoline alkaloids, notably narcotine, could also be derived from reticuline (Morris et al. 2020). Thus, their biosynthesis is intertwined. The subdued synthesis of phenolics notably, Moscatilin, Coumarins, Herniarin, and Hydroxyhydroquinone in the infected samples justify that they are being bypassed towards the synthesis of alkaloids and phenanthrenes. The in vitro raised plants are likely to use these alkaloids to resist the invasion of *Rhizobium*. Hence, we had marginal infection phenotype in the form of hairy roots. L-phenylalanine and L-tyrosine are pivotal precursors for Moscatilin biosynthesis as well. Since infected samples exhibited low amino acid synthesis, it eventually affected the synthesis of stilbenoid pathways which is why the Moscatilin concentration was below the limit of detection in *Rhizobium* infected *D. ovatum* tissues (Fig. 4). Moscatilin and stilbenoids could serve as precursors for the papaverine biosynthetic pathway, which can again serve as an additional cause for the absence of Moscatilin in the infected samples.

On the other hand, Lyciumins are cyclopeptides that function as antimicrobial agents. They encompass cytotoxic, antimicrobial, and enzyme inhibitory activities (Pomilio et al. 2006). Lyciumin B is an inhibitor of both Angiotensin-Converting Enzyme (ACE) and renin; comprising an N-terminal pyroglutamic acid and a macrocyclic bond crosslinking tryptophan-indole nitrogen with a glycine α-carbon. As per a study, it has been detected in the plant tissue after *Rhizobium* infiltration (Kersten and Weng 2018). For every transformation study, the literature has recommended exogenous supplementation of acetosyringone (Cooper and Long 1994). Three different concentrations (100 µM, 150 µM and 200 µM) were used to check the efficiency. The comparison was made with sets without the application of acetosyringone. There was barely any difference in the hairy root appearance (in terms of fresh weight) and the nodulations. Additionally, the acetosyringone added callus turned slimy and vitrescent in 7–8 days. There could be apprehensions and concerns regarding the addition of Zeatin, as cytokinin hinders hairy root formation. But the literature has found that *Rhizobium meliloti* infection in alfalfa which had plasmid, could constitutively secrete trans Zeatin promoted hairy root via root nodulations (Cooper and Long 1994). It has been reported elsewhere that *Agrobacterium tumefaciens* encodes an isopentenyl transferase *that* catalyses the biosynthesis of two naturally occurring cytokinins, isopentenyl-adenine and Zeatin (Akiyoshi et al. 1987; Frébortová and Frébort 2021; Li et al. 2021; Nenadić and Vermeer 2021). The trans zeatin production was shown to be produced both by *Agrobacterium* and *Pseudomonas*, where the highest quantity reported were 44 µM and 1.5 mg l^−1^ (Akiyoshi et al. 1987). *Agrobacterium rhizogenes* strains produced trans-zeatin in the culture at 0.5 to 44 µg/l (Akiyoshi et al. 1987). In our study, Zeatin addition was inevitable to evoke a response from *D. ovatum* tissues concerning somatic embryogenesis and organogenesis. *R. rhizogenes* infection was successfully performed in *Crocus sativus* (Sharma et al. 2021). The new revolution anticipated in hairy root transformation is the development of hairyCRISPR, where genome editing is coupled with transformation in *Oryza sativa* (Kiryushkin et al. 2021). The study uses rice translational enhancer *OsMac3* and the fluorescent protein DsRed1 encoding gene.

The *Rhizobium* infection in the *D. ovatum* tissues appears to be relatively superficial. However, the primary infection by *Rhizobium rhizogenes* could have possibly induced nodulations (Oldroyd et al. 2011), and ‘rol genes’ probably had little role to play. During the nodule inception mechanism, the *Rhizobium* initially recognises the phenylpropanoid in the plant and secretes a rhizobial signalling factor entitled ‘Nod factor’ (Perret et al. 2000). Plant hosts identify these ‘Nod factors’ with their cognate receptors. ‘Nod factor’ recognition triggers plant cell division inducing nodule primordia formation, leading to plant organogenesis (Kawaharada et al. 2015). This phenomenon is under-studied as the mechanism reported is diverse and is host plant-specific. The incidence has been reported even in non-nodulating plant species (Harris and Pitzschke 2020). Some produce determinate nodulation initiating from the root’s inner cortical cells, as witnessed in *Lotus japonica*, whereas in *Medicago truncatula,* indeterminate nodulation was seen originating from the pericycle of the root tissue (Harris and Pitzschke 2020). Bacterial entrapment causes varied manifestations of root hairs. Cell lysis and exocytosis are noticed, which brings about hairy, fluffy root appearances, as witnessed in *Dendrobium ovatum* in the present study. Nodulations has been witnessed in soybean due to *Rhizobium* infection (Rehman et al. 2022). Orchids do entertain mycorrhizal connections, which is essential for their natural environment. But colonisation by *Rhizobium rhizogenes* is less investigated. Occurrences of bacteria in orchids, notably nitrogen-fixing species, are relatively less; if the soil could supply ample nutrients. But in epiphytic orchids, occurrences of bacterial colonisation cannot be ignored. There are several reports on bacteria colonising orchid roots, notably in *Dendrobium moschatum,* such as *Rhizobium* species*, Azotobacter*, *Bacillus radicola* invading roots, causing nodulations (Jakubska-Busse et al. 2021). It is seen that monocots can develop nodules probably if their nodule inception like proteins gets expressed, and these proteins are products of nodule inception genes, which have evolved from nodulating dicots (Liu and Bisseling 2020). In the present study, the SEM analyses showed nodules and restricted infection in *Dendrobium ovatum* root tissues, as noticed in other reports (Abe et al. 1998; Fedorova et al. 2021). The restrictive nodulation and colonisation of *Rhizobium rhizogenes* could also be an escape mechanism of bacteria from the plant defensins. Orchids, unlike legumes, are non-nodulating species, and the bacteria associated with their rhizosphere were less explored (Jakubska-Busse et al. 2021) as the prime focus was majorly on mycorrhizal associations. In vitro co-culture of *Dendrobium ovatum* and *Rhizobium rhizogenes* is a complete model system for decrypting the coadjuvant and combative role of bacterial species with orchids and their mycorrhizae.

In conclusion, the platform presented in this research investigation can be used to derive new insights regarding plant-pathogen interactions and plant defence mechanisms during *R. rhizogenes*-mediated transformation in monocot plants. It can also provide new understandings of how the plant metabolites are routed for defence purposes during *R. rhizogenes* infection phase. For the first time, the present study reports the presence of two novel metabolites in tropical orchid *D. ovatum*, viz., Laudanosine, which serves as a precursor of plant defensins (Bhambhani et al. 2021) and Lyciumin B, that functions as a naturally occurring antimicrobial cyclopeptide (Roumani et al. 2021). The synthesis of defence metabolites could have hampered hairy root production, delimiting the infection towards the peripheral zones of the tissues. It is plausible that phenolics and stilbenoids like Moscatilin could serve as precursors for phenanthrenes. In the case of fungal biotroph infection, it has been noticed that there is an assortment of plant defence compounds that curb the pathogen invasion (Westrick et al. 2021). Limitation of hairy root culture in monocot like *Dendrobiums*, in terms of low biomass of the transgenic roots, can be fixed through the usage of synthetic plasmids (T-DNA binary vectors) in the future. These plasmids are small, and they have suitable selectable markers along with translational enhancers, which might play a significant role in improving the transgene expression. Stilbenoids serve as inducible plant defensins. They operate during the entry of pathogens and during stress such as frost, drought and UV (Eungsuwan et al. 2021). The biosynthetic pathway of Moscatilin is not proven. The genome sequence of *Dendrobium ovatum* is yet to be unravelled. A transcriptome analysis would give a better understanding of the pathway. Among the well-known stilbenoids, Resveratrol stands foremost. It was demonstrated to possess a phytoalexin potential against bunch rot pathogen *Botrytis cinerea* curbing its growth and proliferation. Resveratrol has an oxidising effect on phytoalexin by secreting an enzyme laccase, BcLCC2 (Park et al. 2021). A more straightforward demonstration of stilbenoid detoxification can be witnessed in spruce pathogen *Endoconidiophora polonica*. After being infected into a spruce tree by its bark-beetle vector, the host responds to fungal invasion by producing antifungal compounds, notably ‘Astringin’ and Flavan-3-ol Catechin (Westrick et al. 2021). Moscatilin being structurally similar to Resveratrol, how it effectively helps combat pathogens (both bacterial and fungal) could be presumed from the present study. Thus, the current research investigation serves as a prelude in understanding the metabolite routing mechanisms during infection, enhancing the plant defensins.

## Supplementary Information

Below is the link to the electronic supplementary material.

Supplementary file1 (DOCX 36 KB)

## Data Availability

Experimental data can be available from the corresponding author on reasonable request.
