# Supplementary material for: Rhizobium rhizogenes infection in threatened Indian orchid Dendrobium ovatum mobilises ‘Moscatilin’ to enhance plant defensins
Source: 3 Biotech. 2022 Apr 23;12(5):119. doi: 10.1007/s13205-022-03180-9 (PMC9035196; doi:10.1007/s13205-022-03180-9)
Supplement: Supplementary file 1 — Supplementary file1 (DOCX 36 KB) [file 13205_2022_3180_MOESM1_ESM.docx]

**Supplementary information**

**Table S1.** The exclusive primary metabolite detected in infected and transformed callus-derived plantlets of *Dendrobium ovatum*.

| **Primary metabolite** | **Theoretical m/z** | **Experimental m/z** | **ΔPPM** | **Chemical formula** | **Compound class** | **Biochemical pathway** |
| --- | --- | --- | --- | --- | --- | --- |
| Lyciumin B | 449.1981 | 449.2 | 4 | C44H52N10O11 | Cyclic peptide | unknown |

**Table S2.** The exclusive secondary metabolite detected in infected and transformed callus-derived plantlets of *Dendrobium ovatum*.

| **Secondary metabolite** | **Theoretical m/z** | **Experimental m/z** | **ΔPPM** | **Chemical formula** | **Compound class** | **Biochemical pathway** |
| --- | --- | --- | --- | --- | --- | --- |
| Laudanosine | 358.2013 | 358.20214 | 2 | C21H27NO4 | Isoquinoline alkaloid | Papaverine biosynthesis |

**Table S3.** List of common primary metabolites detected in both infected and non-infected callus-derived plantlets of *Dendrobium ovatum*.

| **Primary metabolite** | **Theoretical m/z** | **Experimental m/z** | **ΔPPM** | **Chemical formula** | **Compound class** | **Biochemical pathway (s)** |
| --- | --- | --- | --- | --- | --- | --- |
| L-Phenylalanine | 166.0863 | 166.08419 | 12 | C9H11NO2 | Amino acid | Phenylpropanoid biosynthesis |
| L-Isoleucine | 132.1019 | 132.10047 | 10 | C6H13NO2 | Amino acid | [Jasmonoyl-amino acid conjugates biosynthesis I](https://pmn.plantcyc.org/PLANT/NEW-IMAGE?type=PATHWAY&object=PWY-6220) and II |
| L-Arginine | 175.1190 | 175.11714 | 10 | C6H14N4O2 | Amino acid | [L-*N^δ^*-acetylornithine biosynthesis](https://pmn.plantcyc.org/PLANT/NEW-IMAGE?type=PATHWAY&object=PWY-6922), Urea cycle |
| Phenylacetic acid | 137.0597 | 137.05825 | 10 | C8H8O2 | Monocarboxylic acid | Phenylalanine metabolism |
| L-Valine | 118.0863 | 118.08486 | 11 | C5H11NO2 | Amino acid | [Indole-3-acetate inactivation V](https://pmn.plantcyc.org/PLANT/NEW-IMAGE?type=PATHWAY&object=PWY-5788) and  [VIII](https://pmn.plantcyc.org/PLANT/NEW-IMAGE?type=PATHWAY&object=PWY-6219), γ-glutamyl cycle |
| Phenylacetaldehyde | 138.0913 | 138.08961 | 12 | C8H8O | Aromatic aldehyde | Phenylethanol biosynthesis |
| N-Carbamoyl-L-aspartic acid | 177.0506 | 177.05218 | 8 | C5H8N2O5 | Amino acid derivative | Unknown |
| L-Proline | 116.0706 | 116.06908 | 13 | C5H9NO2 | Amino acid | L-*N^δ^*-acetylornithine biosynthesis, L-citrulline biosynthesis |
| 4-Guanidinobutanoic acid | 146.0924 | 146.09071 | 11 | C5H11N3O2 | Gamma amino acid | Unknown |
| L-2-Aminoadipic acid | 162.0761 | 162.07468 | 8 | C6 H11 N O4 | Amino acid | Lysine degradation II and III |
| Homoarginine | 189.1346 | 189.13377 | 4 | C7H16N4O2 | Amino acid | Unknown |
| L-Malic acid | 157.0107 | 157.0105 | 1 | C4H6O5 | Beta hydroxy acid | C4 photosynthetic carbon assimilation cycle, Gluconeogenesis I, TCA cycle |
| L-Tyrosine | 182.0812 | 182.07877 | 13 | C9H11NO3 | Amino acid | (*S*)-reticuline biosynthesis I, *Amaryllidacea* alkaloids biosynthesis |
| HNHA (Histone Deacetylase Inhibitor VI) | 304.1366 | 304.13546 | 3 | C17H21NO2S |  | Histone acetylation (regulation of biological activity of Histone Deacetylase) |
| 2-Formylaminobenzaldehyde | 150.0550 | 150.05629 | 8 | C8H7NO2 | Benzaldehyde | Tryptophan metabolism, Indole degradation to anthranil and anthranilate |
| 6-Acetamido-3-aminohexanoate | 189.1234 | 189.12071 | 14 | C8H16N2O3 | β-amino acid | Unknown |
| L-Glutamate | 148.0604 | 148.05877 | 11 | C5H9NO4 | Amino acid | Ammonia assimilation cycle I and II, Inorganic and Organic Nitrogen Assimilation |
| N-Acetyl-L-glutamate 5-semialdehyde | 174.0761 | 174.07366 | 13 | C7H11NO4 | N-acyl-L-alpha-amino acid | L-arginine biosynthesis II (acetyl cycle), L-ornithine biosynthesis I |
| 4-Guanidinobutanoic acid | 146.0924 | 146.09042 | 13 | C5H11N3O2 | Gamma amino acid and derivative | Unknown |
| Succinic anhydride | 101.0233 | 101.02229 | 10 | C4H4O3 | Dicarboxylic acids and derivative | Unknown |
| Hexanethioic acid S-propyl ester | 175.1151 | 175.1165 | 7 | C9H18OS | Fatty acyl thioester | Lipid metabolism |

**Table S4. List of common secondary metabolites detected in both infected and non-infected callus-derived plantlets of *Dendrobium ovatum*.**

| **Secondary metabolite** | **Theoretical m/z** | **Experimental m/z** | **ΔPPM** | **Chemical formula** | **Compound class** | **Biochemical pathway** |
| --- | --- | --- | --- | --- | --- | --- |
| Coumarin | 147.0441 | 147.04244 | 10 | C9H6O2 | Phenylpropanoid | C[oumarin metabolism (to Melilotic acid)](https://pmn.plantcyc.org/PLANT/NEW-IMAGE?type=PATHWAY&object=PWY-5319) |
| Trigonelline | 138.0550 | 138.05335 | 11 | C7H7NO2 | Alkaloid | Nicotinate and nicotinamide metabolism |
| Indoleacrylic acid | 205.0972 | 205.09509 | 10 | C11H9N O2 | Indole | Phenylalanine and Tyrosine metabolism |
| 2-Hexylbenzothiazole | 220.1154 | 220.11545 | 0 | C13H17NS | [Benzothiazoles](http://classyfire.wishartlab.com/tax_nodes/C0000311) | Unknown |
| Herniarin | 177.0546 | 177.05248 | 12 | C10H8O3 | Coumarin | [Simple coumarins biosynthesis](https://pmn.plantcyc.org/PLANT/NEW-IMAGE?type=PATHWAY&object=PWY-5868) |
| 1,4-Dideoxy-1,4-Imino-D-Arabinitol | 134.0812 | 134.07949 | 12 | C5H11NO3 | Pyrrolidine alkaloid | Glycogenolysis |
| Brassilexin | 175.0324 | 175.03471 | 12 | C9H6N2S | Indole (antifungal phytoalexin) | Detoxification |
| Hydroxyhydroquinone | 127.0390 | 127.03736 | 12 | C6H6O3 | Phenol (Benzenetriol) | Unknown |
| Castanospermine | 190.1074 | 190.10532 | 10 | C8H15N O4 | Indolizidine alkaloid | Inhibitor of alpha-galactosidase enzyme |
| Calomelanol G | 433.1282 | 433.12883 | 1 | C25H20O7 | Flavonoid | Unknown |
| Pyrogallin | 205.0495 | 205.04901 | 2 | C11H8O4 | Benzotropolone containing compound | ATP-Competitive Inhibitor of Janus Kinase (JAK) 3 |
| p-Coumaric acid | 165.0546 | 165.05273 | 11 | C9H8O3 | Hydroxycinnamic acid (Phenylpropanoid) | Flavonoid biosynthesis |
| Piperidine | 86.0964 | 86.09562 | 9 | C5H11N | Alkaloid | Piperine biosynthesis |

**Fig. S1. Chromatogram of Moscatilin Standard**

**Fig. S2. Chromatogram of Moscatilin detected in non-infected samples**
